# Engaging private pharmacies to help end TB in India

**DOI:** 10.5588/ijtld.21.0682

**Published:** 2022-05-01

**Authors:** R. Gandhi, K. G. Deepak, G. Verma, S. Chaubey, L. Kumar, J. Klinton, S. Raj, P. Jha, S. Vijayan

**Affiliations:** 1 PATH, Mumbai office, Mumbai, India; 2 Centre for Health Research and Innovation, Joint Effort for Elimination of TB, New Delhi, India; 3 McGill International TB Center, TB PPM Learning Network, Montreal, QC, Canada

Dear Editor,

COVID-19 has negatively impacted progress made towards eliminating TB. This is especially true for India, a country with a high burden of TB, which in 2020 accounted for 41% of the global decrease in case notifications.^1^ India’s private health sector is the first point-of-contact for 51% of patients,^2^ and provides healthcare services to a significant proportion of the population. In 2018, to achieve the End TB goals, the Government of India (GoI), with support from the Global Fund, engaged with the private sector and initiated the Joint Effort for Elimination of TB (JEET). JEET is implemented through patient provider support agencies (PPSAs), which serves as an interface between the National TB Elimination Programme (NTEP) and the private sector healthcare. The project is being implemented in 23 states by a consortium of partners under the guidance of the Central TB Division (CTD) and the Ministry of Health and Family Welfare (MOHFW). The Centre for Health Research and Innovation (CHRI; New Delhi, India), an affiliate of PATH, operates in 31 PPSAs in three states (Uttar Pradesh, Assam and Maharashtra) with five implementation partners, Alert India (New Delhi, India), Maharashtra Janavikas Kendra (MJK; Mumbai, India), World Vision India (Mumbai, India), Mamta (Mumbai, India) and Leprosy Relief Association (LEPRA; Mumbai, India).

TB is a notifiable disease in India and the GoI’s 2018 directive mandates pharmacies, chemists and druggists to notify complete details of TB patients, and the sale of anti-TB drugs in accordance with the prescription of a registered medical practitioner under schedule H1.^3^ Additionally, pharmacies are required to maintain a patient record of such sales. India alone contributes to 24% of the global notification gap attributable to underreporting and under-diagnosis of TB.^1^ To optimise the notification gap observed during surveys in the field, JEET decided to extend the focus to pharmacies. Therefore, CHRI planned to undertake measures to uncover missing TB cases through a pilot intervention focusing on engagement with pharmacies in 13 PPSA districts of Maharashtra.

The pilot for pharmacy engagement began in October 2019 to create awareness and garner support for TB elimination and the GoI mandate to record complete patient data for schedule H1 drugs, particularly anti-TB drugs. Pharmacies were encouraged to share such patient details in Annexure III, a notification format provided in the GoI mandate to capture relevant patient details (including name, age, gender, GoI ID, address, phone number, date of TB diagnosis, date of treatment initiation, date of prescription, name and contact of treating doctor, date of dispensing of medicines, number of days for which medicines dispensed and other details such as reporting pharmacy contact details). Pharmacies were also informed about the Nikshay portal, a web based online notification and patient management system in India. Of the 14,896 pharmacies at the 13 pilot sites, a total of 5,477 (i.e., close to one-third) were engaged. These selected pharmacies were validated by the field staff and were either the in-house pharmacies or the neighbouring ones where TB patients bought the anti-TB drugs prescribed by PPSA-engaged doctors. The end-to-end step-wise process followed during the pharmacy engagement pilot intervention is shown in the Figure. To strengthen pharmacy engagement, support was sought from Food and Drugs Administration (FDA) Maharashtra, which issued a circular encouraging the pharmacies to record and share complete details of TB patients.

Between October 2019 and March 2021, JEET collected information on 32,787 patients. Due to incomplete and incorrect details, information collected for 86% of the patients could not be validated and used. Information on the remaining 14% (i.e., 4,505 TB cases) could be validated and was notified in the system. Of the total 4,505 TB cases notified, the majority were from pilot sites of Aurangabad Municipal Corporation (MC) (*n* = 1,011), followed by Kalyan Dombivili MC (KDMC) (*n* = 792). In spite of the high collection rates of patient information from PPSA districts Thane (*n* = 6,285), Pune (*n* = 3,540), Vasai Virar (*n* = 3,368) and Sholapur (*n* = 3,216), the post-validation and notification yields were as low as 6%, 11%, 7% and 9%, respectively. This reiterates the importance of collecting complete patient information to close the gap on TB notification rates. The pilot site of Miara Bhaynder MC was able to validate the highest percentage of patient information (52%), which contributed to the notification of 561 TB cases. Furthermore, based on the information provided by the pharmacists, CHRI was able to identify 222 new private providers who were not previously engaged in the programme.

Incomplete and incorrect information was a key challenge during the implementation. Such information made it difficult to contact patients to ascertain their notification status and to determine whether drugs were for TB or other ailments. Other challenges included limited staff to conduct regular, needs-based in-person visits, difficulty in identifying pharmacies that stock and dispense anti-TB drugs in the dynamic market scenario, and training of pharmacies on how to use the Nikshay portal for case notification. Additionally, pharmacies were unable to collect all the necessary information during busy, peak hours. Between April–September 2020, the control measures introduced in India for COVID-19, drastically affected the TB programme.^4^–^8^ For our pilot, this led to restricted movement of the field team, staff and patients, and they faced resistance from the pharmacies to share information. However, once services resumed, notifications history and past intelligence was used to categorise and prioritise pharmacy visits to collect data for the lockdown period. This patient information was collated daily to check its completeness and validated by the partner NGO by phone. The TB notifications were then restored.

**Figure:**
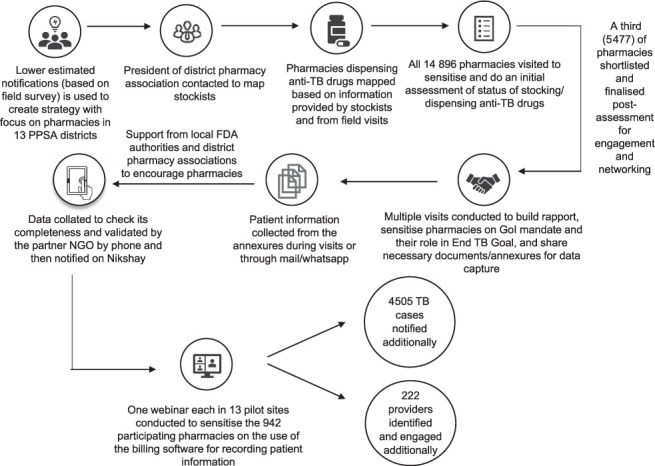
Step-wise process followed during the pharmacy engagement pilot. PPSA =patient provider support agencies; FDA=Food & Drug Administration; NGO = non-governmental organization; GoI = Government of India.

This engagement with pharmacies highlights their importance in boosting TB notifications and as a crucial source of information on private sector patients and providers. By engaging with 5,477 pharmacies out of more than 80,000 registered across the 13 PPSA districts in the state of Maharashtra, JEET was able to notify 4,505 missing cases. Further evaluation on yield and cost-effectiveness of the strategies will help demonstrate that pharmacy engagement initiatives can be a game-changer in identifying the missing millions. CHRI and PATH are exploring various strategies to further engage pharmacies. A pilot intervention for an innovative software solution to capture all relevant patient and provider information and the anti-TB drugs being dispensed at pharmacies is currently being designed. This uses an API (application programming inter-phase), which integrates billing software into Nikshay to generate real-time information.
